# Rare *BLK*, *CEL*, *KLF11*, *PDX1*, and *PAX4* Gene Variants in Russian Patients with Monogenic Diabetes: Clinical and Molecular Characterization

**DOI:** 10.3390/biomedicines13102452

**Published:** 2025-10-09

**Authors:** Rita I. Khusainova, Ildar R. Minniakhmetov, Dmitry N. Laptev, Mariya P. Koltakova, Roman V. Deev, Bulat I. Yalaev, Yaroslav V. Dvoryanchikov, Elena A. Sechko, Natalia G. Mokrysheva

**Affiliations:** Endocrinology Research Centre, Moscow 117292, Russia; khusainova.rita@endocrincentr.ru (R.I.K.); laptev.dmitry@endocrincentr.ru (D.N.L.); koltakova.mariya@endocrincentr.ru (M.P.K.); deev.roman@endocrincentr.ru (R.V.D.); yalaev.bulat@endocrincentr.ru (B.I.Y.); dvoryanchikov.ya@endocrincentr.ru (Y.V.D.); sechko.elena@endocrincentr.ru (E.A.S.); mokrisheva.natalia@endocrincentr.ru (N.G.M.)

**Keywords:** maturity-onset diabetes of the young (MODY), monogenic diabetes, rare MODY subtypes, genetic heterogeneity, pathogenic variants, missense mutation, frameshift mutation

## Abstract

**Background:** Maturity-onset diabetes of the young (MODY) is a heterogeneous group of monogenic diabetes forms that are frequently misclassified as type 1 or type 2 diabetes due to overlapping phenotypic features. The true prevalence of MODY is likely substantially underestimated. As DNA-based diagnostics become increasingly accessible, an expanding number of novel genetic variants are being identified. **Objectives:** The aim of this study was to characterize the clinical and genetic features of patients carrying rare variants in the *BLK*, *KLF11*, *PAX4*, *PDX1*, and *CEL* genes, with attention to population-specific aspects, family history, and treatment outcomes. **Methods:** Targeted next-generation sequencing (NGS) using a custom-designed panel covering 27 genes implicated in MODY, neonatal diabetes, and related hereditary syndromes was performed on the Illumina NovaSeq 6000 platform (Illumina). **Results:** We identified 21 variants in five genes associated with rare MODY subtypes among 24 unrelated patients. MODY9 was diagnosed in two unrelated patients of Russian ethnicity harboring an identical heterozygous missense mutation in exon 5 of the *PAX4* gene (HG38, chr7:127615049G>A, c.191C>T, p.Thr64Ile), which has not been previously described in patients with diabetes. MODY11 was diagnosed in a patient carrying the c.773-1G>A variant in the *BLK* gene. A patient with a de novo c.40_41dupGC (p.Val15Glnfs*41) variant in the *KLF11* gene was clinically diagnosed with type 1 diabetes. **Conclusion:** Our findings expand the current understanding of rare MODY subtypes and contribute to the growing body of evidence on the spectrum and frequency of potentially pathogenic variants in *BLK*, *CEL*, *KLF11*, *PDX1*, and *PAX4* genes across ethnically diverse populations worldwide.

## 1. Introduction

Maturity-onset diabetes of the young (MODY) is the most common type of monogenic diabetes mellitus (DM), with an estimated prevalence of 108 per million individuals [1]. Among children with diabetes, MODY accounts for 1.0–6.5% of cases, depending on the population studied [2]. In European populations, its frequency is approximately 1–5 per 10,000 individuals (or 1 per 23,000 children), whereas in other regions—such as Africa, Asia, the Middle East, or South America—its prevalence remains poorly defined [3]. The true prevalence of MODY is likely substantially underestimated: approximately 80% of individuals with monogenic diabetes are misclassified as having type 1 or type 2 diabetes, probably due to overlapping phenotypic features with these subtypes [4,5]. Diabetes subtypes may also coexist, with distinct pathogenic mechanisms occurring in the same individual, while rarer forms of monogenic diabetes often go unrecognized [6].

The classical diagnostic criteria for MODY include onset of diabetes before age 25, diabetes in at least two consecutive generations, absence of pancreatic β-cell autoimmunity, and preserved β-cell function, defined as either no requirement for insulin therapy or a serum C-peptide level greater than 200 pmol/L after three years of insulin treatment [7]. However, approximately 50% of genetically confirmed MODY cases do not meet these established diagnostic criteria; some patients may present with pancreatic autoantibodies or carry de novo mutations without a family history of diabetes [8].

To date, 14 MODY subtypes have been described, each associated with pathogenic variants in a single gene involved in β-cell differentiation, development, or function. The inheritance pattern is predominantly autosomal dominant. Nearly 90% of MODY cases are caused by variants in glucokinase (*GCK*, MODY2), hepatocyte nuclear factor 1-alpha (*HNF1A*, MODY3), or hepatocyte nuclear factor 4-alpha (*HNF4A*, MODY1), although the distribution of subtypes varies across populations [9].

In a UK cohort, *HNF1A* variants were identified in 52% of probands, followed by *GCK* (32%), *HNF4A* (10%), *HNF1B* (6%), and *NEUROD1* or INS (<1%) [1]. Similarly, in Norway, HNF1A accounted for 53% of MODY cases, *GCK* for 30%, *HNF4A* for 7.5%, and *HNF1B* for 5.6% [10]. In contrast, genetic testing of 272 German and Austrian children with suspected MODY revealed *GCK* mutations in 62% of cases, *HNF1A* mutations in 31%, and HNF4A mutations in 4%. Notably, 17% of mutation carriers tested positive for at least one pancreatic β-cell autoantibody (GAD, IA-2, or IAA), indicating phenotypic overlap between MODY and type 1 diabetes [11].

In India, targeted next-generation sequencing of 10 MODY genes in 56 patients identified potentially pathogenic variants in *HNF4A*, *GCK*, *HNF1A*, *PDX1*, *HNF1B*, *NEUROD1*, and *PAX4* in 11 individuals, suggesting a more complex etiological spectrum in this population [12].

A study of 224 Turkish patients (45% female; mean age at diagnosis 9.4 ± 4.1 years) found the following distribution: *GCK* (65%), *HNF1A* (19%), *HNF4A* (3.6%), *KLF11* (3.6%), and *HNF1B* (3.1%). Twelve rare variants were also detected in *PDX1* (n = 1), *NEUROD1* (n = 3), *CEL* (n = 1), *INS* (n = 3), *ABCC8* (n = 3), and *KCNJ11* (n = 1) [13]. Another Turkish cohort analysis revealed 43 MODY-specific variants absent in controls, including 11 missense and four synonymous substitutions; variants p.D202E (*NEUROD1*), p.R461Q (*KLF11*), p.G248R (*BLK*), and p.S385F (*KCNJ11*) were described for the first time in that study [14].

In our previous work, we reported pathogenic or likely pathogenic variants in 23 genes (*GCK*, *HNF1A*, *HNF1B*, *ABCC8*, *ALMS1*, *WFS1*, *IGF1R*, *PDX1*, *NSD1*, *SH2B1*, *KCNQ1*, *HNF4A*, *INSR*, *KLF11*, *PAX4*, *INS*, *BLK*, *LIPE*, *KCNJ11*, *LMNA*, *ZFP57*, *KDM6A*) in 277 Russian individuals with suspected hereditary diabetes, with the highest number of variants (n = 113) detected in *GCK* [15]. However, some authors have argued that there is insufficient evidence to support a causal role for *BLK*, *KLF11*, or *PAX4* variants in MODY pathogenesis, and these genes should not be routinely included in diagnostic testing [16].

At present, knowledge regarding rare MODY subtypes—such as MODY4 (*PDX1*), MODY7 (*KLF11*), MODY8 (*CEL*), MODY9 (*PAX4*), and MODY11 (*BLK*)—remains scarce. Data on mutation spectra, frequencies, prevalence, and clinical features are limited, making the study of rare MODY variants in diverse populations both relevant and necessary. MODY is frequently misclassified, and reconsideration of an existing diabetes diagnosis may be warranted at any stage [17].

Accurate diagnosis is crucial, as it guides therapeutic decisions, informs prognosis, and enables assessment of disease risk in relatives, given the hereditary nature of MODY.

The aim of this study was to characterize the clinical and genetic features of patients carrying rare variants in the *BLK*, *KLF11*, *PAX4*, *PDX1*, and *CEL* genes, with attention to population-specific aspects, family history, and treatment outcomes.

## 2. Materials and Methods

The present study was based on our previously published cohort of 506 unrelated probands with clinically suspected monogenic diabetes, who all underwent targeted NGS using a custom-designed panel covering 27 genes implicated in MODY, neonatal diabetes, and related hereditary syndromes (see [15] (material and methods)). From this cohort, we selected 24 unrelated probands carrying variants in *BLK*, *CEL*, *KLF11*, *PDX1*, or *PAX4* for detailed genotype–phenotype analysis in the current work. The study protocol included comprehensive clinical evaluation and detailed collection of family history. All participants underwent biochemical testing [venous plasma glucose (fasting or during an oral glucose tolerance test [OGTT]), glycated hemoglobin (HbA1c), C-peptide, and insulin (fasting or during OGTT)] and immunological testing for pancreatic autoantibodies (ICA, GADA, IA-2A, and ZnT8A).

Written informed consent was obtained from all participants or their legal guardians. The study was approved by the Bioethics Committee of the Endocrinology Research Centre, Moscow, Russia (protocol code No.16 dated 13 September 2023). The work was carried out using the materials of the Unique Scientific Facility “Collection of Biological Material from Patients with Endocrine Pathologies” of the Endocrinology Research Center (Moscow, Russia).

Genomic DNA was extracted from peripheral blood lymphocytes obtained from participants using the MagPure Blood DNA Kit (Magen, Guangzhou, China). Quality and quantity assessments of the extracted DNA were performed using a NanoDrop 2000 spectrophotometer (Thermo Fisher Scientific, Waltham, MA, USA) and a Qubit 3.0 fluorometer with the Qubit dsDNA HS Assay Kit (Invitrogen, Carlsbad, CA, USA).

Targeted next-generation sequencing (NGS) was performed on the Illumina NovaSeq 6000 platform (Illumina, San Diego, CA, USA) using paired-end reads (2 × 150 bp). The assay covered the coding sequences of canonical transcripts along with 25 nucleotides of flanking intronic regions. A custom-designed targeted panel included the following genes: *GCG*, *GLUD1*, *WFS1*, *HNF1A*, *GCK*, *INS*, *HNF1B*, *ABCC8*, *HNF4A*, *RFX6*, *PTF1A*, *NEUROD1*, *AKT2*, *ZFP57*, *INSR*, *EIF2AK3*, *PPARG*, *PAX4*, *PDX1*, *GLIS3*, *KCNJ11*, *SLC16A1*, *FOXP3*, *BLK*, *CEL*, *KLF11*, *SCHAD*, and *GCGR*. Library preparation was performed using the KAPA HyperPlus Kit (Roche, Basel, Switzerland) following the manufacturer’s protocol.

NGS data were processed using an automated bioinformatics pipeline, which included read alignment to the human reference genome (GRCh38), post-alignment processing, variant calling, quality filtering, and annotation of all identified variants across all known transcripts based on the RefSeq database. Pathogenicity assessment followed the ACMG/AMP guidelines [18] and employed multiple computational algorithms for variant impact prediction. SpliceAI and AdaBoost were applied to evaluate the potential effects of variants on splice sites and adjacent intronic regions. Clinical significance was assessed using OMIM and HGMD. The potential impact of variants on protein structure and function was evaluated using Annovar, SIFT, MutationTaster, and MutPred, in conjunction with population databases including the 1000 Genomes Project, Exome Aggregation Consortium (ExAC), dbSNP, and HGMDB, among others [18].

All identified variants were validated by Sanger sequencing using primers specific to the target exon regions, performed on an ABI 3500 Genetic Analyzer (Thermo Fisher Scientific, USA). For confirmed variants, parental testing was conducted to determine inheritance patterns or de novo origin.

## 3. Results

A total of 21 variants in five genes—*BLK*, *CEL*, *KLF11*, *PAX4*, and *PDX1*—were identified in 24 patients. These genes have been implicated in various subtypes of diabetes, including MODY; however, published data provide conflicting evidence regarding the contribution of pathogenic variants in these genes to hereditary diabetes.

In most patients, elevated blood glucose levels were first detected during childhood, raising suspicion of a hereditary form of diabetes. Clinical and laboratory data, together with family histories, were analyzed and correlated with the identified genetic variants and relevant literature. Seven of the 21 detected variants were absent from all public databases and are reported here for the first time. Six of these novel variants showed high pathogenic potential according to in silico analyses (Table 1). For one previously undescribed synonymous variant in *KLF11* (c.393A>G; p.Lys131=), splicing prediction algorithms indicated a potential role in alternative splicing.

**Variants in the *BLK* Gene**

In the *BLK* gene (NM_001715.3), three missense variants previously listed in databases and one novel splice-site variant in exon 8 (HG38, chr8:11556657G>A, c.773-1G>A) in a heterozygous state were identified, with a sequencing depth of 134×. This variant is predicted to disrupt the canonical acceptor splice site, resulting in a loss-of-function allele, consistent with reports that *BLK* deficiency impairs β-cell function and may contribute to diabetes pathogenesis [19]. In the patient carrying the c.773-1G>A variant, severe hyperglycemia (25 mmol/L) was documented at age 8 years, along with zinc transporter autoantibodies (ZnT8A: 33.0 U/mL). A family history of type 1 diabetes on the maternal side was noted, although no genetic testing was performed in relatives (Table 2). The presence of autoimmune markers in rare MODY forms has been previously reported [13]. The low level of single islet antibody could not exclude a diagnosis of MODY11 in this patient (Table 2).

The missense *BLK* variants identified in our cohort were absent from ClinVar, had extremely low allele frequencies in gnomAD v4.1.0, and were predicted to be likely pathogenic by multiple computational algorithms (Table 1). Clinical and laboratory features varied among patients carrying these variants. In patient #1 (Table 2), low level of anti-insulin autoantibody (IAA: 18.7 U/mL) were observed, along with hyperinsulinemia without a corresponding increase in C-peptide levels, a normal BMI, and no clinical signs of hyperinsulinism. Notably, the patient’s mother died at a young age from diabetic nephropathy secondary to type 1 diabetes. Two other patients with *BLK* variants were autoantibody-negative. In patient #3, hyperglycemia at diagnosis was 15.8 mmol/L, with a C-peptide level of 0.41 ng/mL; subsequent follow-up demonstrated progressive β-cell failure leading to complete loss of function. The patient’s mother carried the same variant but had no carbohydrate metabolism disorders. Patient #4 was overweight and had insulin resistance (HOMA-IR: 10.7) with a positive family history; his mother carried the same variant and had a history of gestational diabetes requiring insulin therapy.

The *BLK* Ala71Thr mutation was first reported in 2009 in six MODY families, leading to the recognition of MODY type 11 [19]. Our findings are consistent with previous reports that BLK-MODY exhibits heterogeneous clinical presentations. Several studies have noted the frequent coexistence of overweight or obesity—acknowledged as important contributors to hyperglycemia—together with relatively low serum insulin levels and other metabolic abnormalities [20]. To date, 17 pathogenic variants in *BLK* have been reported; however, the role of this gene as a definitive cause of MODY remains to be fully clarified [21].

**Variants in the *CEL* Gene**

We identified two frameshift variants in the carboxyl ester lipase (*CEL*) gene—a single-nucleotide deletion c.391delG (p.Met131Trpfs64) and a single-nucleotide duplication c.2139dup (p.Val714Argfs6)—as well as a missense substitution p.Arg164His (c.491G>A) in three unrelated patients of Russian ethnicity. Variants in *CEL* cause CEL-associated maturity-onset diabetes of the young type 8 (CEL-MODY, MODY8), a very rare disorder most often resulting from frameshift variants within the proximal segment of the CEL variable number tandem repeat (VNTR) region [22]. This condition typically manifests with diabetes before the age of 25 years, slowly progressive exocrine pancreatic insufficiency, pancreatic lipomatosis, and cyst formation.

In patient #7, the c.2139dup (p.Val714Argfs*6) variant was located within the *CEL* VNTR region; however, at the age of 9 years, there was no evidence of exocrine pancreatic insufficiency. This variant is listed in the Human Genetic Variation Database (rs768557807), has an allele count of 1 in 58,230, and has not been reported in the scientific literature as causative of a disease phenotype.

All three patients carrying *CEL* variants tested negative for pancreatic β-cell autoantibodies. A family history of type 2 diabetes mellitus (T2DM) was present in patients #5 and #7, and type 1 diabetes mellitus (T1DM) in patient #6, all on the paternal side (Table 2). DNA testing of affected relatives was not possible due to unavailability of biological samples. All three patients developed diabetes in childhood (before 11 years of age), and none showed evidence of exocrine pancreatic dysfunction.

In patient #5, overweight was documented at age 15 years, with daily blood glucose levels ranging from 7.0 to 9.5 mmol/L and HbA1c values between 5.2% and 6.6% during follow-up. The patient required no insulin therapy and maintained glycemic control within the target range by adhering to dietary recommendations.

Patient #6 exhibited a gradual decline in β-cell function, progressing to complete loss of function within 5 years of diabetes onset. Additionally, two heterozygous variants were identified in the cystic fibrosis transmembrane conductance regulator (*CFTR*) gene: exon 3 c.220C>T (p.Arg74Trp; rs115545701; previously reported; likely pathogenic) and exon 15 c.2563G>A (p.Val855Ile; rs397508397; HGMD:CM094117; previously reported; variant of uncertain significance). Biallelic *CFTR* variants cause cystic fibrosis (OMIM #219700), in which cystic fibrosis-related diabetes (CFRD) develops in 40–50% of affected adults, characterized by both insulin deficiency and insulin resistance [23]. Neonatal screening for cystic fibrosis was negative, and no pancreatic cystic lesions were detected. It is plausible that, in this patient, the combined effects of the three identified variants in two genes contributed to β-cell loss.

In patient #7, overweight and fasting hyperglycemia were observed at age 13 years; however, HbA1c values were within the normal range, and an oral glucose tolerance test (OGTT) revealed no impairment of glucose tolerance, although moderate insulin resistance was present.

The absence of exocrine pancreatic dysfunction in probands, the lack of confirmed parental variants, and the young age of the patients do not provide sufficient evidence to support a definitive diagnosis of MODY8. Nevertheless, our findings expand the spectrum of reported *CEL* variants with potential pathogenicity in the Russian population and contribute to the growing database of variants in this gene. The possibility of developing carbohydrate metabolism disorders later in life cannot be excluded; all patients remain under clinical surveillance.

**Variants in the *KLF11* Gene**

We also identified four variants of potential clinical significance in the Kruppel-like factor 11 (*KLF11*) gene, which regulates metabolic pathways, is highly conserved from Drosophila to humans, and plays a role in insulin secretion [24]. Altered KLF11 function has been associated with maturity-onset diabetes of the young type 7 (MODY7) and neonatal diabetes; however, the precise molecular mechanisms remain incompletely understood.

**Figure 1:**
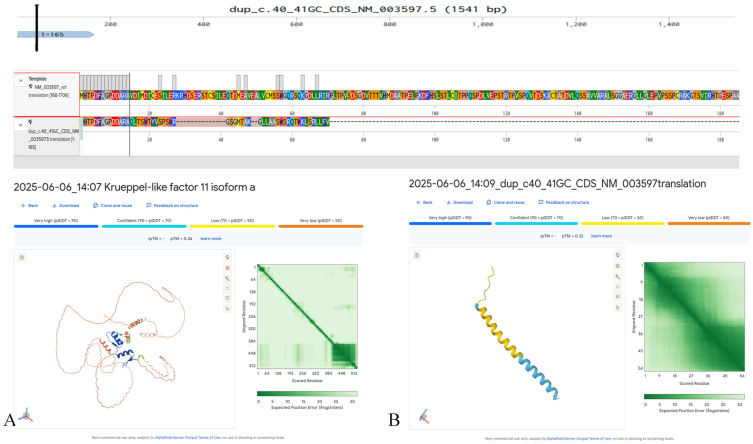
Predicted structural impact on the KLF11 protein (NM_003597.5) of the c.40_41dupGC (p.Val15Glnfs41) variant, generated in silico using AlphaFold3. (**A**) Predicted structure of the wild-type KLF11 protein. (**B**) Predicted altered structure resulting from the p.Val15Glnfs41 frameshift variant.

In exon 1 of *KLF11* (NM_003597.5), we detected a heterozygous c.40_41dupGC (p.Val15Glnfs41) variant that has not been previously described in the literature and is absent from major population databases (e.g., gnomAD, ExAC) (Figure 1). This variant likely results in complete loss of function and protein degradation due to severe structural disruption. The patient, a 16 year old diagnosed with diabetes at age 10 years, tested positive for all pancreatic β-cell autoantibodies, meeting the diagnostic criteria for T1DM (Table 2). Family history included T2DM in the maternal grandfather; both parents were unaffected. The c.40_41dupGC (p.Val15Glnfs41) variant in *KLF11* was not detected in parental DNA, consistent with a de novo occurrence.

There is growing evidence that pancreatic autoantibodies may also be present in forms of diabetes other than type 1. In a Czech MODY cohort, autoantibodies to glutamic acid decarboxylase (GAD) or islet antigen-2 (IA-2) were detected in 25% of cases, and a positive autoantibody status was reported in 17% of patients in a series from Germany and Austria [25].

In our cohort, patient #10 (Table 2) carrying the *KLF11* c.514G>A (p.Gly172Arg) variant tested positive for both GAD and IA-2 autoantibodies. At the age of 13 years, she underwent pylorus-preserving pancreaticoduodenectomy for a solid pseudopapillary neoplasm of the pancreatic head. One month postoperatively, fasting plasma glucose was 13.5 mmol/L, with a C-peptide level of 1.57 ng/mL. Two years later, C-peptide had declined to 0.01 ng/mL. Both parents were unaffected. Given the patient’s young age at onset, genetic testing was performed and revealed the p.Gly172Arg missense variant in *KLF11*. While MODY8 is characterized by progressive pancreatic dysfunction and cyst formation, the presence of autoantibodies, absence of a family history, low predicted pathogenicity, and relatively high population frequency of this variant make a diagnosis of MODY7 unlikely. The patient remains under clinical surveillance. The identified variant was previously described in a 22-year-old patient from China as likely pathogenic in MODY. The variant is found in the gnomAD v3.1.2 population database in the heterozygous state with a frequency of 0.0006570% (1 heterozygote) [26].

In two unrelated Russian patients (patients #11 and #12), we identified the *KLF11* c.1447C>T (p.Pro483Ser; rs201735305) variant. Both had a family history of diabetes: in one case, the father had type 1 diabetes mellitus (T1DM) and the paternal grandfather had type 2 diabetes mellitus (T2DM); in the other, the mother and a sibling had T1DM without autoantibodies, and the maternal grandfather had T2DM. DNA testing was not performed in affected relatives. Despite carrying the same variant, the two patients differed in age at onset, and the second patient tested positive for GAD autoantibodies (Table 2). Neither patient was overweight, and during dietary therapy, time-in-range was 86%, with postprandial hyperglycemia occurring only during dietary non-adherence. The c.1447C>T variant results in a non-conservative amino acid substitution; three of five in silico prediction tools indicated a potentially damaging effect on protein function. However, its allele frequency (6.3 × 10^−8^) is more consistent with a likely benign classification.

**Figure 2:**
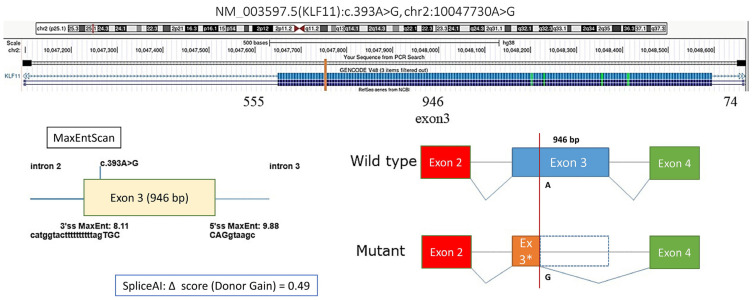
Prediction of the effect of the *KLF11* NM_003597.5:c.393A>G variant on splicing, * Author’s drawing.

We also identified a synonymous *KLF11* variant, c.393A>G (p.Lys131=), with a moderate predicted probability of creating a novel alternative donor splice site (donor gain score: 0.46), potentially leading to skipping of 863 bp within exon 3 and altering the resulting protein sequence (Figure 2). In patient #9, fasting hyperglycemia was observed, with postprandial testing revealing impaired glucose tolerance. The patient was not overweight, and HbA1c during follow-up ranged from 6.3% to 7.12%. The same variant was detected in his mother, who had a history of gestational diabetes mellitus (GDM). In both cases, adherence to dietary therapy resulted in improved glycemic control; the mother maintained dietary restrictions during pregnancy but returned to a regular lifestyle postpartum.

Taken together, patients in our cohort carrying *KLF11* variants demonstrated considerable heterogeneity in clinical presentation. While MODY7 could not be confidently diagnosed in any of them, these cases are presented due to their rarity and the limited literature available on *KLF11* variants in individuals with diabetes mellitus.

**Variants in the *PAX4* gene**

While MODY7 could not be confidently diagnosed in any of them, these cases are presented due to their rarity and the limited literature available on *KLF11* variants in individuals with diabetes mellitus.

In the *PAX4* gene (NM_001366110.1), we identified five missense variants not previously reported in the literature–p.Thr64Ile (c.191C>T), p.His128Pro (c.383A>C), p.Arg155Gln (c.464G>A), p.Val214Met (c.640G>A), and p.Ala330Val (c.1013C>T)—as well as one variant affecting a donor splice site (HG38, chr7:127611942T>C, c.771+3A>G). The p.Thr64Ile (c.191C>T) variant was found in two unrelated patients of Russian ethnicity.

In exon 5, we identified a novel heterozygous variant (HG38, chr7:127615049G>A, c.191C>T) in an 8-year-old female proband, resulting in a p.Thr64Ile substitution, with a sequencing read depth of 375×. This variant is absent from the gnomAD population frequency database, lies in a conserved position, and is recorded in ClinVar (rs2535520513) without phenotypic annotation and is classified as a variant of uncertain significance (VUS). Computational algorithms predicted a deleterious effect on protein function. The patient tested negative for diabetes-associated autoantibodies. Both her mother (T2DM onset at age 26) and father (T2DM onset at age 36) had diabetes. The proband’s disease presentation met classical MODY9 criteria (polyuria, polydipsia, fatigue, easy fatigability), with fasting plasma glucose of 11.7 mmol/L. DNA testing revealed that the variant was inherited from her mother.

The same variant was found in a 12-year-old female with negative autoantibodies, whose mother was diagnosed with T2DM at age 26, was initially treated with metformin for 1.5 years, and later switched to insulin therapy. Given the family history and similar clinical features, both patients were diagnosed with MODY9.

**Variants in the *PDX1* gene**

In exon 1 of *PDX1* (NM_000209.4), we identified the missense variant c.383A>C (p.His128Pro); in exon 2, three additional variants were found: c.719C>G (p.Pro240Arg; rs753881947), c.533A>C (p.Glu178Ala), and c.417C>G (p.Tyr139Ter), all in the heterozygous state, with the last variant (p.Tyr139Ter) identified in two unrelated patients.

The c.383A>C (p.His128Pro) variant was recently reported in an adult Chinese patient during screening of a type 2 diabetes cohort. Functional analysis using a dual-luciferase reporter assay demonstrated significantly reduced transcriptional activity of the *INS* promoter compared to wild-type *PDX1*, supporting classification as likely pathogenic [27]. In our cohort, the variant was detected in a 5-year-old patient with fasting plasma glucose of 11.0 mmol/L, polyuria, polydipsia, and weight loss; autoantibodies were negative. The patient’s mother had no evidence of carbohydrate metabolism disorders, and the father was unavailable for testing.

The *PDX1* c.417C>G (p.Tyr139Ter) variant, identified in two unrelated patients, resulted in disease onset at age 15 in both cases, with negative autoantibodies (Table 2). One patient had no family history, while the other had maternal lineage diabetes.

The *PDX1* c.533A>C (p.Glu178Ala) variant was reported by Ribeiro AF et al. (2024) [28] in a 4-year-old girl from Portugal with no family history, hyperglycemia, HbA1c of 13%, and C-peptide <0.1 ng/mL. Our patient with the same variant, negative for autoantibodies, developed diabetes at age 11 with fasting glucose of 9.9 mmol/L, urine glucose “++”, ketones “+”, HbA1c of 6.0%, and OGTT results of 6.3 mmol/L (30 min), 14.9 mmol/L (60 min), and 8.1 mmol/L (120 min). C-peptide was 1.95 ng/mL and showed good glucose-stimulated C-peptide secretion; HbA1c was 6.6%. OGTT indicated impaired glucose tolerance. Both parents were unaffected, consistent with the Portuguese case.

In patient #24 with the *PDX1* c.719C>G (p.Pro240Arg) variant, a stage II (T2 N0 M0) solid pseudopapillary tumor of the pancreatic tail was diagnosed, and a spleen-preserving distal pancreatectomy with splenic vessel resection (Warshaw procedure) was performed.

In summary, we identified variants in *BLK*, *CEL*, *KLF11*, *PDX1*, and *PAX4* that may contribute to various forms of diabetes, including rare MODY subtypes. Our study demonstrated marked clinical variability among patients with mutations in the same gene, and in most cases, a definitive MODY diagnosis could not be established. Nevertheless, MODY9 was diagnosed in two unrelated Russian patients carrying the same novel heterozygous missense mutation in exon 5 of *PAX4* (HG38, chr7:127615049G>A, c.191C>T, p.Thr64Ile), and MODY11 in one patient with the *BLK* c.773-1G>A variant. Type 1 diabetes was diagnosed in the patient with a de novo c.40_41dupGC (p.Val15Glnfs*41) mutation in *KLF11*.

## 4. Discussion

The available literature presents conflicting data on the clinical significance of variants in the *BLK*, *CEL*, *KLF11*, *PDX1*, and *PAX4* genes in various forms of diabetes mellitus (DM).

In our cohort, we identified four probands carrying BLK variants, including a novel splice-site variant (c.773-1G>A) and three missense changes predicted to be likely pathogenic. By domain context, these variants localize to the SH2 region or the adjacent kinase domain, which are functionally important for BLK-mediated signaling in β-cells. The splice-site variant was associated with severe early-onset diabetes (diagnosed at age 8 years, blood glucose 25 mmol/L), while the missense variants demonstrated heterogeneous clinical presentations ranging from autoantibody-negative diabetes to insulin resistance and family history of gestational diabetes. These observations highlight both the potential involvement of BLK in β-cell dysfunction and the challenge of interpreting such variants due to incomplete penetrance and the coexistence of autoimmune markers in some patients.

In 2009, Borowiec et al. demonstrated that *BLK*—a non-receptor tyrosine kinase of the Src proto-oncogene family—is expressed in pancreatic β-cells, where it enhances glucose-stimulated insulin synthesis and secretion via upregulation of the transcription factors *PDX1* and *NKX6*.1. They reported that the p.Ala71Thr mutation substantially impaired this function, concluding that *BLK* represents a previously unrecognized modulator of β-cell function, whose deficiency may lead to maturity-onset diabetes of the young type 11 (MODY11) [19]. However, a subsequent study (2013) by Bonnefond et al. sequenced *BLK* in 64 French and Danish patients with a MODY phenotype and screened for p.A71T in 4901 individuals with type 2 diabetes (T2DM) and 4280 controls. No nonsynonymous *BLK* mutations were identified in MODY phenotype cases, while p.A71T was present in 52 controls. The authors concluded that high-penetrance MODY due to p.A71T is unlikely, although the variant may confer an increased risk of T2DM in individuals with obesity [29].

More recently, a case was reported of a 17-year-old female presenting with polyuria, polydipsia, and weight loss over two months, with a family history of diabetes (father diagnosed at age 23, paternal grandfather and aunt also affected). Laboratory results showed a blood glucose level of 299 mg/dL, HbA1c of 15.4%, anti-GAD antibodies at 2.67 IU/mL, IAA at 2.38 IU/mL, C-peptide at 1.22 ng/mL, insulin at 8.25 μU/mL, urine ketones ++, and urinary glucose ++. A heterozygous BLK variant c.1081G>T (p.Gly361Trp) was identified [30].

To date, only a small number of MODY11 cases have been described, and further research is required to clarify the role of *BLK*. It is plausible that different *BLK* variants may contribute not only to monogenic diabetes but also to increased susceptibility to polygenic forms. Therefore, *BLK* should not be excluded from diagnostic gene panels, and systematic collection of variant spectra, frequency data, and genotype–phenotype correlations should be continued.

In our cohort, we identified three *CEL* variants of potential clinical significance: two frameshift changes (p.Met131Trpfs64 and p.Val714Argfs6) and one missense substitution (p.Arg164His). Importantly, the p.Val714Argfs6 variant lies within exon 11/VNTR, the region classically associated with toxic gain-of-function and the MODY8 phenotype, whereas p.Met131Trpfs64 and p.Arg164His are located outside the VNTR, where pathogenicity is less established and may reflect haploinsufficiency or uncertain effects. All three probands were 9–13 years old and showed no evidence of exocrine pancreatic dysfunction at the time of evaluation, consistent with the reported age-dependent expressivity of CEL-MODY and highlighting the importance of longitudinal follow-up.

MODY8, caused by mutations in the carboxyl ester lipase (*CEL*) gene, is characterized by pancreatic lipomatosis and exocrine pancreatic dysfunction and results from dominant frameshift mutations in the VNTR region (exon 11). It is hypothesized to represent a protein misfolding disorder, with a toxic gain-of-function effect of mutant proteins in pancreatic tissue [31]. This subtype of MODY was first described by Raeder et al. in adults (mean age 36 ± 10 years), with exocrine pancreatic dysfunction, including low fecal elastase levels [32]. MODY8 is extremely rare, with approximately eight pathogenic variants reported to date. The phenotype may vary by genotype, particularly depending on whether the mutation lies within the VNTR region; no mutations outside the VNTR have been associated with exocrine dysfunction, and in such cases symptoms are confined to diabetes [33].

In our study, the c.2139dup (p.Val714Argfs*6) variant lies within the *CEL* VNTR region; however, in our 9-year-old patient, no exocrine pancreatic dysfunction was observed. A Japanese case report described a CEL mutation in exon 2 (c.146_147delCT; p.Ser49CysfsTer52) in a 13-year-old girl presenting with her first episode of diabetic ketoacidosis (DKA) and impaired insulin secretion. Insulin secretion recovered within two months of initiating insulin therapy, and no treatment was required for the next two years. DKA recurred at age 15, after which she has been maintained on insulin therapy. This mutation lies outside the VNTR, yet exocrine dysfunction was observed, suggesting that such features may develop later in life [33].

All our patients with likely pathogenic *CEL* variants were aged 9–13 years and did not exhibit exocrine pancreatic dysfunction. This observation aligns with a Turkish report describing a 6-year-old patient with CEL-MODY, random hyperglycemia, no family history of diabetes, negative autoantibodies, and an HbA1c of 5.6%. At diagnosis, blood glucose was 137 mg/dL, C-peptide 0.7 ng/mL, and the patient was managed with dietary therapy alone [13]. This case is comparable to our patient #6 with c.2139dup (p.Val714ArgfsTer6), detected during fasting blood tests, with normal HbA1c, normal OGTT, and no treatment initiated. These findings underscore the need for longitudinal follow-up and the potential for next-generation sequencing (NGS)-based studies to expand knowledge of CEL-MODY genotype–phenotype correlations across age groups and populations.

MODY7, caused by mutations in the *KLF11* gene, is extremely rare and appears to be more common in Asian populations. In our cohort, we identified five probands carrying KLF11 variants: a novel frameshift mutation (c.40_41dupGC, p.Val15Glnfs*41), a missense variant (c.514G>A, p.Gly172Arg), a recurrent missense variant (c.1447C>T, p.Pro483Ser) in two unrelated patients, and a synonymous variant (c.393A>G, p.Lys131=) predicted to affect splicing. Structurally, the frameshift affects the N-terminal regulatory region, p.Gly172Arg lies within the central transactivation domain, and p.Pro483Ser maps to the C-terminal zinc-finger DNA-binding region, all functionally important for KLF11’s role as a transcription factor regulating the INS promoter. The clinical presentations were heterogeneous: some patients exhibited autoimmune markers consistent with type 1 diabetes, while others had impaired glucose tolerance or a positive family history of diabetes. None of the cases allowed a confident diagnosis of MODY7, but these observations illustrate the phenotypic diversity of KLF11 carriers and the difficulty of establishing causality in the absence of segregation data.

MODY7 was first reported in patients with early-onset T2DM carrying two rare variants (p.Ala347Ser and p.Thr220Met) [34]. Subsequent studies identified additional rare *KLF11* variants in MODY screening cohorts: p.His418Gln, associated with early-onset type 1B diabetes in a Japanese cohort [35]; p.Lys453del [36]; and p.Ile89Leu, p.Gly484Ser [1], and p.Cys354Phe [37] in Chinese populations.

A heterozygous *KLF11* variant (c.1045C>T, p.Pro349Ser) was reported in a Chinese proband, his mother, maternal grandmother, and elderly aunt, although the latter two were unaffected. In silico analyses suggested an amino acid side-chain alteration within transcriptional regulatory domain 3, and luciferase reporter assays confirmed impaired INS promoter activity. In vitro studies further demonstrated disrupted insulin secretion from pancreatic β-cells [38].

A recent large-scale Chinese study identified three novel missense *KLF11* variants (p.Gly172Arg, p.Glu265Lys, and p.Gly251Glu) [26]. None of the probands had islet autoantibodies, and the mean age at diagnosis was 26.3 years. The p.Gly172Arg variant was also found in our patient #9, diagnosed at age 13, with positive GAD and IA-2 antibodies—differing from the Chinese case, which had a four-generation family history of diabetes.

Another report described a 30-year-old male from China and his unaffected mother carrying a heterozygous *KLF11* c.793G>A (p.Glu265Lys) mutation [39]. The authors proposed that this variant may demonstrate incomplete penetrance for glucose intolerance, supporting the view that routine inclusion of KLF11 in MODY genetic testing may not be warranted.

In a Turkish cohort, KLF11-MODY accounted for 3.5% (n = 8) of MODY cases. The mean age at diagnosis was 11.2 ± 2.0 years. Three patients had random hyperglycemia, seven presented with diabetic symptoms, and three had diabetic ketosis. Family history was positive in all cases. At diagnosis, mean blood glucose was 278 ± 175 mg/dL, C-peptide 2.2 ± 2.0 ng/mL, and HbA1c ranged from 5.6% to 10.2%. Only one patient had positive anti-GAD antibodies. Treatments varied: dietary management (n = 1), oral antidiabetic drugs (n = 1), oral therapy plus basal insulin (n = 2), and intensive insulin therapy (n = 4) [13].

Given the low prevalence of MODY7, its prolonged subclinical course, and incomplete penetrance, the association between *KLF11* variants and disorders of glucose metabolism has been questioned [40]. However, recent data on the spectrum and prevalence of different *KLF11* variants in patients with hyperglycemia suggest that it is premature to draw definitive conclusions regarding the limited role of this gene in the development of diabetes, including hereditary forms.

In our cohort, we identified four *PDX1* variants: p.His128Pro, p.Tyr139* (two unrelated probands), p.Glu178Ala, and p.Pro240Arg. Structurally, p.His128Pro lies in the N-terminal transactivation region, p.Tyr139* is a truncation immediately proximal to the homeodomain (thereby removing the DNA-binding region), p.Glu178Ala falls within the homeodomain, and p.Pro240Arg is C-terminal to the homeodomain. These positions provide a strong mechanistic rationale for MODY4 in carriers of p.Tyr139* and p.Glu178Ala (loss of DNA binding/transactivation), while p.His128Pro is supportive but less definitive, and p.Pro240Arg remains a VUS given its location outside the homeodomain and limited corroborating evidence.

Pancreatic and duodenal homeobox 1 (PDX1) functions as a transcriptional activator for multiple pancreatic genes and plays a crucial role in both pancreatic development and β-cell function [41]. In humans, heterozygous *PDX1* mutations are frequently associated with maturity-onset diabetes of the young type 4 (MODY4) [42], whereas homozygous mutations typically result in neonatal diabetes mellitus (NDM) [43]. Globally, *PDX1* variants segregating with monogenic diabetes (PDX1-MODY) have been reported in only a few families, and due to their rarity, the clinical characteristics of affected individuals remain poorly defined [44].

Many patients continue to be misdiagnosed with type 2 diabetes (T2DM) because of the broad phenotypic spectrum, despite MODY4 and NDM typically manifesting within the first two years of life. In China, MODY4 accounts for 0.59% of early-onset T2DM cases. The same study reported the missense variant c.383A>C (p.His128Pro), which we also identified in our patient #18. Functional analysis using a dual-luciferase reporter assay demonstrated that H128P markedly reduced *INS* promoter transcriptional activity compared with wild-type PDX1 [27].

The p.Glu178Lys (*PDX1*) variant detected in our study has also been reported in a Portuguese patient [44], where it was shown to increase PDX1 protein degradation and reduce its transcriptional activity [45]. Similarly, the p.Pro33Ala variant, located in the highly conserved transactivation domain, has been classified as likely pathogenic and associated with MODY [46]. Another variant at the same amino acid position (p.Pro33Thr) was shown to decrease binding to the *INS* promoter and reduce transcriptional activity in vitro, leading to a MODY phenotype [47]. Our findings therefore add to the limited number of reported PDX1-MODY cases and support the pathogenicity of variants affecting the homeodomain, while highlighting the uncertainty of those located outside this critical region.

In our cohort, we found six rare *PAX4* variants: p.Thr64Ile (two unrelated probands), p.Arg155Gln, p.Thr213Met, p.Val214Met, p.Ala330Val, and c.771+3A>G (donor-site region). Structurally, p.Thr64Ile resides in the paired DNA-binding domain, p.Thr213Met and p.Val214Met localize to the homeodomain, p.Arg155Gln maps to the inter-domain linker between Paired and Homeodomain, and p.Ala330Val lies in the C-terminal regulatory tail. Given the critical roles of the paired and homeodomain in β-cell lineage specification and maintenance, variants within these domains (particularly p.Thr64Ile) have high biological plausibility for MODY9, consistent with our two familial cases. By contrast, variants in linker or C-terminal regions and splice-adjacent changes remain of uncertain significance in the absence of segregation or functional confirmation.

The paired box gene 4 (*PAX4*; OMIM167413), the MODY9 gene, encodes a transcription factor that plays a pivotal role in the development of β- and δ-cells. PAX4 is involved in the differentiation of pancreatic β- and δ-cell precursors during early development and in the maintenance of β-cell identity thereafter [48]. Several *PAX4* variants have been linked to type 1 diabetes (T1DM) [49], type 2 diabetes (T2DM) [50], ketosis-prone type 2 diabetes (KPD) [51], and monogenic diabetes, first reported in two Thai patients [52].

More than a decade after the initial report, only a few additional studies—mainly in Asian populations—have confirmed the involvement of *PAX4* mutations in monogenic diabetes. Age at onset in MODY9 cases ranges from 14 to 50 years. Patients carrying the same mutation may present with markedly different phenotypes, ranging from isolated fasting hyperglycemia or impaired glucose tolerance to classic diabetic symptoms (polyuria, polydipsia, fatigue) or even diabetic ketoacidosis.

A missense mutation p.Arg164Gln (c.491G>A) was recently described in a large Brazilian family, segregating with diabetes and showing heterogeneous clinical features and treatment responses; age at diagnosis ranged from 24 to 50 years [53]. Another study identified a missense mutation (c.487C>T, p.Arg163Trp) in exon 7 of *PAX4* in a 19-month-old child and his father. This substitution, replacing a polar amino acid with a non-polar one, was predicted to impair the inhibitory activity of *PAX4* on the *INS* and GCG promoters, leading to hyperglycemia and diabetes. The proband was diagnosed with MODY9, whereas the father was normoglycemic at the time, possibly due to epigenetic modifiers or incomplete penetrance [54].

Management of MODY9 generally parallels that of other MODY types, with lifestyle interventions (diet, physical activity) and insulin therapy when indicated. Some patients respond to oral hypoglycemic agents, and treatment should be tailored according to disease progression. Outcomes vary widely: some patients exhibit only mild glucose intolerance, while others develop severe complications such as nephropathy, retinopathy, or even death due to end-stage renal disease.

MODY9 is a rare subtype caused by mutations in the transcription factor PAX4, which contains two DNA-binding domains—Paired and Homeo. *PAX4* expression in humans is tissue-specific, and alternatively spliced mRNA transcripts encode protein isoforms differing in their N- and C-terminal regions. Functional studies have shown that full-length *PAX4* with intact DNA-binding domains has maximal activity in a transient expression system using a firefly luciferase reporter under the control of the *INS* promoter in HEK293 cells. Transcriptional activity is markedly reduced in variants lacking eight N-terminal amino acids and/or with truncations of the C-terminal Homeo domain [55].

In animal models (mouse Pax4), knockout results in complete absence of β-cells, a marked increase in glucagon-producing α-cells, and death within days of birth. Loss-of-function *PAX4* mutations in humans are implicated in diabetes pathogenesis. In adults, *PAX4* expression is restricted to a subpopulation of β-cells capable of proliferating in response to increased metabolic demand. Upregulation of *PAX4* promotes β-cell survival and proliferation, while ectopic expression of PAX4 in α- or δ-cells can generate functional β-like cells, improving glucose regulation in experimental diabetes models. Thus, PAX4 is a promising therapeutic target for β-cell protection and regeneration in diabetes [56].

In our study, we identified 21 variants in *BLK*, *CEL*, *KLF11*, *PDX1*, and *PAX4*. Several *PDX1* and *PAX4* variants, particularly those affecting the homeodomain and paired domain, showed strong biological plausibility for MODY4 and MODY9, and were classified as likely pathogenic. In contrast, *BLK* and *KLF11* variants demonstrated incomplete penetrance, frequent coexistence of autoimmune markers, or occurrence in unaffected relatives, which limited their clinical interpretation. For *CEL*, frameshift variants were consistent with a possible MODY8 mechanism, but exocrine manifestations were not observed at the time of evaluation. Family segregation analyses could be performed only in a minority of cases and were frequently uninformative, precluding definitive classification. As a result, several variants were categorized as variants of uncertain significance (VUS). Taken together, these findings highlight both the potential contribution and the limitations of interpreting rare variants in these genes, which explains the conflicting impact observed across our cohort.

## Figures and Tables

**Table 1:** Prevalence frequencies and clinical significance according to ACMG criteria and in silico pathogenicity assessment of identified variants.

Gene	cDNA, Variant	Protein, Variant	ACMG Classification	ID	Allele Frequency (gnomAD v4.1.0)	CADD	Individual Predictions (Varsome) *		
							Pathogenic	Uncertain	Benign
*BLK*	c.717G>T	p.Gln239His	VUS	rs778299790	1.239 × 10^−6^	22.6	0	6	25
	c.773-1G>A	-	LP	-	not described	34.0	9	4	0
	c.803T>C	p.Ile268Thr	VUS	-	8.893 × 10^−6^	26.4	10	11	4
	c.1033G>A	p.Ala345Thr	VUS	rs1300783845	1.24 × 10^−6^	26.3	6	14	2
*CEL*	c.391delG	p.Met131Trpfs*64	LP	-	not described	-	-	-	-
	c.491G>A	p.Arg164His	VUS	rs778299790	3.718 × 10^−6^	27.4	15	4	0
	c.2139dup	p.Val714Argfs*6	LP	rs768557807	1.49 × 10^−5^	19.4	-	-	-
*KLF11*	c.40_41dupGC	p.Val15Glnfs*41	LP	-	not described	-	-	-	-
	c.393A>G	p.Lys131=	VUS	-	not described	8.79	-	-	-
	c.514G>A	p.Gly172Arg	VUS	rs1351414401	3.098 × 10^−6^	18.84	0	9	22
	c.1447C>T	p.Pro483Ser	VUS	rs761563032	5.08 × 10^−5^	22.8	3	10	11
*PAX4*	c.191C>T	p.Thr64Ile	LP	rs2535520513	not described	27.3	24	4	0
	c. 464G>A	p.Arg155Gln	VUS	-	5.391 × 10^−5^	17.31	0	4	22
	c.638C>T	p.Thr213Met	VUS	rs528075802	0.00003	27.2	8	3	2
	c.640G>A	p.Val214Met	VUS	-	6.815 × 10^−5^	23.8	9	5	6
	c.771+3A>G	-	VUS	rs776955589	4.957 × 10^−6^	21.4	1	1	0
	c.1013C>T	p.Ala330Val	VUS	-	5.349 × 10^−4^	10.1	0	6	25
*PDX1*	c.383A>C	p.His128Pro	LP	-	9.925 × 10^−6^	30.0	10	10	3
	c.417C>G	p.Tyr139*	LP	-	not described	35.0	0	3	5
	c.533A>C	p.Glu178Ala	LP	-	not described	31.0	18	6	0
	c.719C>G	p.Pro240Arg	VUS	rs753881947	7.257 × 10^−6^	9.01	3	3	25

For CADD (Combined Annotation Dependent Depletion), we classified variants based on the phred-like score, with a threshold of 20, below which variants were classified as benign and otherwise harmful, as suggested by the authors. * AlphaMissense, BLOSUM, BayesDel, addAF, BayesDel, noAF, DANN, DEOGEN2, EIGEN, EIGEN-PC, EVE, FATHMM, FATHMM-MKL, FATHMM-XF, LIST-S2, LRT, M-CAP, MVP, MaxEntScan, MetaLR, MetaRNN, MetaSVM, MitImpact, MitoTip, MutPred, MutationAssessor, MutationTaster, PROVEAN, Polyphen2-HDIV, Polyphen2-HVAR, PrimateAI, REVEL, SIFT, SIFT4G, phastCons100way, vertebrate, phyloP, scSNV-ADA, scSNV-RF (https://varsome.com/about/resources/germline-implementation/#insilicopredictions, accessed on 13 September 2023); NA—Variant not found in gnomAD, P—Pathogenic, LP—Likely Pathogenic, LB—Likely Benign, B—Benign.

**Table 2:** Clinical, genetic and laboratory characteristics of patients.

Patients	Genetic Variant	Diabetes History (Years)	Age of Manifestation	* HbA1c	** Plasma Glucose mmol/L	*** Autoantibodies U/mL	C-Peptide pmol/L	HOMA	Family History
1	*BLK*: c.717G>T, p.Gln239His	0.6	15.4	5.4	10	IAA (18.7)	2.39	18.4	mother died, no info about T1D, maternal uncle T1DM
2	*BLK*: c.773-1G>A	3.5	8	7.6	25	ZnT8A (33.04)	1.21	1.31	mother T1DM, maternal grandfather T1DM
3	*BLK*: c.803T>C, p.Ile268Thr	4.9	9.8	9	15.8	negative	0.01	-	mother *BLK*: c.803T>C, p.Ile268Thr
4	*BLK*: c.1033G>A, p.Ala345Thr	6	9.6	6.18	5.8	negative	4.01	10.7	mother GDM (*BLK*: p.Ala345Thr), maternal grandmother T2DM
5	*CEL*: c.391delG, p.A131fs	1.7	11	6.2	5.2	negative	2.92	2.7	father and paternal grandmother T2DM
6	*CEL*: c.491G>A, p.R164H *CFTR*: c.220C>T, p.R74W (rs115545701)/c.2563G>A, p.V855I (rs397508397)	9.4	5.5	5.9	5.3	negative	0.01	-	father T1DM (*CEL*: c.491G>A, p.R164H)
7	*CEL*: c.2139dup, p.Val714ArgfsTer6	0.6	8.6	5.4	6.1	negative	2.04	3.3	father and paternal grandmother T2DM
8	*KLF11*: c.40_41dupGC, p.Val15GlnfsTer41	7	10.3	8.9	14.4	IA-2A—81.6 GADA—44.6 ZnT8A—72.9	0.438	-	maternal grandfather DM
9	*KLF11*: 393A>G, pLys131=	4.8	8	6.9	6	negative	0.31	-	mother GDM *KLF11*: 393A>G, p.Lys131=, father T1DM
10	*KLF11*: c.514G>A, p.Gly172Arg	4.7	13.1	5.8	6.3	GAD—12.6 IA2 –58.1	1.57	-	paternal grandmother DM
11	*KLF11*: c.1447C>T, p.Pro483Ser	6	8.9	6.6	7.8	negative	2.67	-	father T1DM and paternal grandfather T2DM
12	*KLF11*: c.1447C>T, p.Pro483Ser	1.4	21	5.7	7.74	GAD—298.7	1.28	-	Mother DM?, brother T1DM (no high-risk antibodies or HLA were detected), maternal grandfather T2DM
13	*PAX4*: c.191C>T, p.Thr64Il	5	12.7	6.4	13.5	negative	1.4	5.9	mother *PAX4*: c.191C>T, p.T64I
14	*PAX4*: c.191C>T, p.Thr64Il	0.1	8.7	7.8	11.4	negative	3.8	-	mother and father T2DM *PAX4*: c.191C>T, p.Thr64Ile (mother)
15	*PAX4*: c. 464G>A, Arg155Gln	1.7	2.3	13.9	26.5	IA2 – 23	0.124	-	father *PAX4*: c. 464G>A, p.R155Q
16	*PAX4:* c.638C>T), p.(Thr213Met)	19	14	-	20	negative	0.88	-	father T1DM, brother T2DM
17	*PAX4:* c.640G>A, p.Val214Met	0.1	0.3	7.6	13.8	negative	0.65	-	no heredity for diabetes
18	*PAX4*: c.771+3A>G	0.1	10.6	7.3	6	negative	1.05	-	no heredity for diabetes
19	*PAX4*: c.1013C>T, p.Ala330Val	0.10	4.5	5.2	4.6	negative	-	-	Mother and maternal grandfather T2DM
20	*PDX1*: c.383A>C, p.His128Pro	3.6	5.3	9.1	11	negative	0.72	-	no carbohydrate metabolism disorders of the mother
21	*PDX1*: c.417C>G, p.Tyr139Ter	0.1	15.2	7.9	9.1	negative	0.68	-	no heredity for diabetes
22	*PDX1*: c.417C>G, p.Tyr139Ter	0.5	15.6	5.8	6.2	negative	2.08	4.0	Mother–T1DM (insulin 0.3 U/kg/d (no genetic test result) and maternal grandmother T2DM (pills)
23	*PDX1*: c.533A>C, p.Glu178Ala	3.1	11.4	6	9.9	negative	1.87	1.26	maternal grandfather DM
24	*PDX1*: c.719C>G, p.Pro240Arg	2.10	15.1	8.1	17	negative	0.34	-	Parents died, no data on diabetes

* Glycated hemoglobin level at the time of diabetes manifestation, ** plasma glucose level at the time of diabetes manifestation, *** autoantibodies for type 1 diabetes at the manifestation. Type 1 diabetes (T1DM), gestational diabetes (GDM), diabetes mellitus type 2 (T2DM), diabetes mellitus unspecified (DM).

## Data Availability

The data from this study can be obtained from the corresponding author upon making a reasonable request if there are no privacy or ethical issues.
